# Comparison of the Efficacy of Two Different Local Anesthesia Techniques for Mesotherapy in Temporal Region Alopecia

**DOI:** 10.7759/cureus.58498

**Published:** 2024-04-17

**Authors:** Sharanika A Nagaja, Rubin S John, Santhosh P Kumar, Murugesan Krishnan

**Affiliations:** 1 Oral and Maxillofacial Surgery, Saveetha Dental College and Hospitals, Saveetha Institute of Medical and Technical Sciences, Saveetha University, Chennai, IND

**Keywords:** innovative technique, novel therapies, platelet-rich plasma (prp), local analgesia, post operative pain, hair regrowth, mesotherapy

## Abstract

Introduction

Mesotherapy is a wide range of minimally invasive injections. In mesotherapy, a mixture of various tonics is injected into the skin. These include plant extracts, various medications, vitamins, enzymes, hormones, growth factors, and other factors that will help treat alopecia. Most commonly, a mixture of platelet-rich plasma (PRP) and vitamins is used. In mesotherapy treatment for hair regrowth in the temporal region, zygomaticotemporal nerve blocks, supratrochlear nerve blocks, and supraorbital nerve blocks are given. The zygomaticotemporal nerve, supraorbital nerve, and supratrochlear nerve are the branches of the trigeminal nerve. They provide sensation on the lateral side of the forehead, which is the temple region.

Methods

A sample size of 100 people was taken for the study who were undergoing mesotherapy in the bilateral temporal region for alopecia. Each group had 50 subjects: group I was given supratrochlear, supraorbital, and zygomaticotemporal nerve blocks as local anesthesia techniques, and group II was given supratrochlear and supraorbital nerve blocks. PRP was injected using an insulin syringe. Pain was assessed using a visual analog scale (VAS).

Results

During the procedure, a mean VAS of 1 and 3 was observed in groups I and II, respectively, during the procedure (p-value 0.023). A mean VAS of 3 and 5 was observed in groups I and II, respectively, after three hours (p-value 0.000). This shows a significant difference in the pain experienced by the subjects between the groups.

Conclusion

This study proves that the zygomaticotemporal nerve, used along with supratrochlear and supraorbital nerve blocks, is better at producing analgesia and reducing pain.

## Introduction

Mesotherapy is a cutting-edge medical procedure that dates back to the late 20th century. It has become a popular, non-invasive way to treat a wide range of medical and cosmetic issues. It involves the microinjection of medications and/or vitamins into the middle layer of the skin. Dr. Michel Pistor, a French physician, described it [1]. When he administered intravenous procaine to an asthmatic patient, he discovered that the patient's hearing had improved. He subsequently began experimenting with subcutaneous procaine injections for a variety of purposes, and in 1976 he came up with the term "mesotherapy." Subsequently, it has been attempted to treat numerous additional ailments, including tinnitus, eczema, and joint pain. The phrase "treatment of the mesoderm" was originally used to refer to mesotherapy [2]. 

This novel treatment entails injecting a specially prepared mixture of vitamins, minerals, amino acids, and pharmaceutical and homeopathic remedies straight into the mesoderm, the middle layer of skin [3]. Delivering therapeutic chemicals precisely to the targeted locations to promote cellular regeneration and therapeutic benefits is the basic idea underlying mesotherapy [4]. Mesotherapy has a broad range of uses in the medical and cosmetic domains. Mesotherapy has shown promise in treating a range of musculoskeletal disorders, including sports injuries, osteoarthritis, and chronic pain [5]. Compared to conventional oral drugs, it has fewer systemic side effects and can administer pharmaceuticals directly to damaged tissues, improving efficacy. Furthermore, mesotherapy has shown promise in the management of alopecia and cellulite, among other dermatological issues [6].

Mesotherapy has become more well-known in the beauty field as a non-surgical method of skin regeneration and anti-aging. Mesotherapy promotes the creation of collagen and elastin by injecting a mixture of skin-nourishing ingredients straight into the dermal layer, improving the texture, tone, and firmness of the skin [7]. This method is often used to target particular regions, like the face, neck, and décolletage, and it provides people with a non-invasive substitute for conventional cosmetic operations [8]. New developments in mesotherapy have made it possible to provide more specialized and focused care. In order to promote tissue regeneration and repair, the patient's own platelets are extracted and concentrated as part of a specialized mesotherapy called platelet-rich plasma (PRP) therapy [9]. Platelet-rich plasma (PRP) injections have historically targeted important facial areas supplied by the supratrochlear and supraorbital nerves. On the other hand, a fascinating recent advancement is the addition of the zygomaticotemporal block to these injections [10].

Supratrochlear nerve (STN) and supraorbital nerve (SON) injections are well-known for their potency in platelet-rich plasma (PRP) therapy for revitalizing the forehead and surrounding areas of the eyes. To block the supratrochlear nerve (STN), insert 1 to 2 cc of lidocaine, bupivacaine, or a combination of the two just above the eyebrow across its medial boundary using the needle. Using the same puncture as for the supratrochlear nerve (STN), an injection of 1 to 2 cc of anesthetic can be made here to anesthetize the supraorbital nerve (SON), which is situated approximately 2 cm lateral to the supratrochlear nerve, or it can be made laterally [8,11]. However, these injections might not be sufficient to address issues in the occipital and temporal regions [12]. The zygomaticotemporal block has been added as an additional strategy to get around this restriction. In order to provide a more thorough and all-encompassing therapy approach, PRP is injected into the zygomaticotemporal nerve, which innervates the temporal region as well as the occipital region [13,14].

By reaching areas that conventional injection sites would overlook, the zygomaticotemporal block has drawn attention to its capacity to improve platelet-rich plasma (PRP) therapy's overall outcomes [14,15]. By adding this extra step, healthcare professionals can give their patients a more customized, comprehensive treatment that addresses a range of concerns. This invention perfectly captures the dynamic character of mesotherapy, as practitioners and researchers work to further develop and broaden the application of this ground-breaking medicinal and cosmetic procedure [16].

Having everything taken into account, mesotherapy is a fantastic medical development with uses ranging from pain relief to facial rejuvenation. It has been a popular choice for people looking for efficient and customized solutions because of its capacity to administer tailored therapies with little intrusiveness. The incorporation of the zygomaticotemporal block into platelet-rich plasma (PRP) therapy is an example of how the medical community is dedicated to improving mesotherapy methods so that patients can receive the most complete and advantageous care [16-18]. Since there is currently no evidence to support combining these blocks with other mesotherapy procedures, the purpose of this study was to evaluate the efficacy of zygomaticotemporal blocks in addition to supratrochlear and supraorbital blocks for mesotherapy in the temporal area. The primary objective of this study is to assess the pain experienced during and after three hours of the procedure using the above-mentioned nerve blocks.

## Materials and methods

Study design and setting

The Oral and Maxillofacial Surgery Department of Saveetha Dental College, Saveetha Institute of Medical and Technical Sciences (SIMATS) University, Chennai, India, conducted this prospective comparative study from June 2022 to May 2023. The Saveetha University Institutional Ethics Committee gave its approval for this work (IHEC/SDC/OMFS-2203/23/344).

Inclusion and exclusion criteria

With Norwood Grades 2 and 3, every patient had a history of diffuse hair thinning and loss. Patients between the ages of 18 and 40 who needed platelet-rich plasma (PRP) therapy for hair growth were included. Individuals who refused to participate in the trial, had systemic illnesses or were allergic to local anesthesia were eliminated from the study. 

Sampling

The sample size of 100 patients was determined using G power with the aid of a study conducted by Gentile et al. (2018) [19], taking into account α = 5% and a power of 95. In this prospective comparative study, where the allocation ratio is one to one, the patients were split into two groups: group I received regional blocks consisting of supratrochlear, supraorbital, and zygomaticotemporal nerve blocks, while group II received regional blocks consisting of supratrochlear and supraorbital nerve blocks. Following the eligibility assessment, patients were randomly selected using the block randomization method and divided into two groups.

Procedure

**Figure 1:**
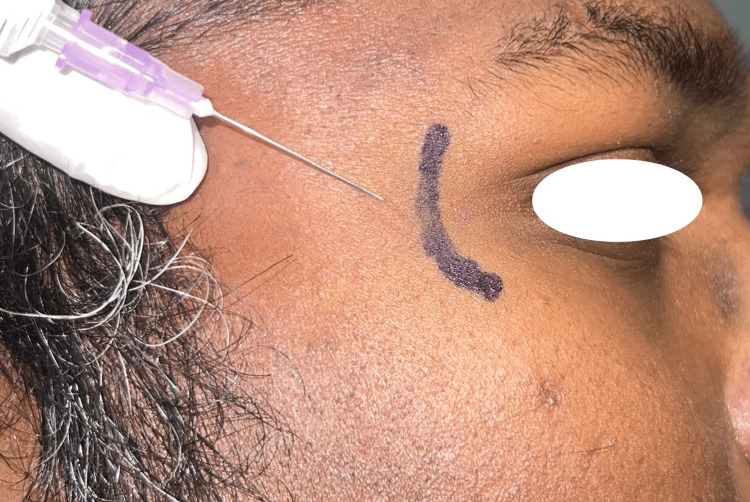
Landmarks for zygomaticotemporal nerve block.

Following an explanation of the process, the patients were given an appointment. One radial vein was used to extract ten milliliters of blood for each group. Centrifuging the blood for ten minutes at 2400 rpm and fifteen minutes at 3600 rpm. The group I received a regional nerve block consisting of supratrochlear, supraorbital, and zygomaticotemporal nerve blocks (landmarks of supratrochlear nerve block above the eyebrow across its medial boundary; supraorbital nerve block: 2 cm lateral to supratrochlear nerve block; zygomaticotemporal nerve block: 15 mm posterior to the frontozygomatic suture and 22 mm above the zygomatic arch) (Figure 1). The area was anesthetized with 2% lignocaine mixed with 1:80000 adrenaline, and platelet-rich plasma (PRP) was injected from the anterior hairline to the scalp's vertex, excluding the occipital and temporal regions.

The patients in group II received the same dosage of local anesthetic by a supratrochlear, supraorbital regional nerve block (same technique as group I). With the exception of the kind of block prescribed for local anesthesia, the same treatment was administered in both groups. It takes seven to nine minutes for the effects of regional anesthesia to take effect, and two injections are needed for each side. During the operation and three hours following the treatment, participants' levels of pain and analgesia resulting from the nerve blocks were evaluated using the visual analog scale (VAS). Patients reported pain was measured using a visual analog scale (VAS) [15]. The visual analog scale uses face emojis to represent a 10-point Likert scale with equal intervals, with 0 representing no pain and 10 representing severe pain.

Statistical analysis

IBM SPSS Statistics for Windows, Version 24 (Released 2016; IBM Corp., Armonk, New York, United States) was used for statistical analysis. The median and percentage of the VAS scores were evaluated using descriptive statistics. Using the Shapiro-Wilk test, the normalcy test was evaluated. To evaluate the variations in pain between before and after surgery, the Wilcoxon sign rank test was used. The Mann-Whitney U test was utilized to evaluate the variations in pain scores among the injection methods with a significance level of less than 0.05 (p<0.05). 

## Results

**Figure 2:**
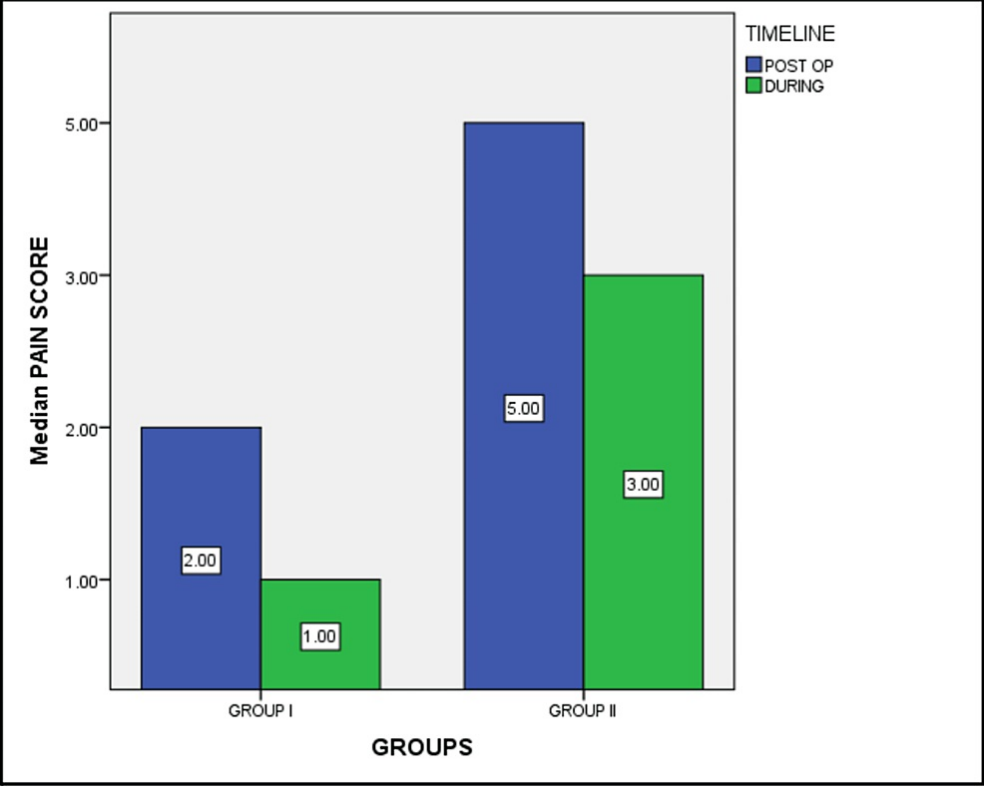
Median pain score among both the groups during and post the PRP procedure PRP: platelet-rich plasma

The study assessed the effectiveness of zygomaticotemporal nerve blocks used in platelet-rich plasma (PRP) treatment for hair growth. This prospective comparative study had 100 patients who participated in the study and were divided into groups I (n=50) and II (n=50), and the pain was scored using the visual analog scale (VAS), which is expressed as ordinal data. The median pain score of group I during the procedure was 1, and three hours after the procedure, it was 2. Similarly, the median pain score of group II during the procedure was 3, and after the procedure was 5 (Figure 2). The individual score distribution with percentages is given in Table 1.

**Table 1:** Distribution of visual analog scale (VAS) pain scores among both the groups during and post the platelet-rich plasma (PRP) procedure

VAS Scores	During the procedure		Post 3 hours of procedure	
	Group I n (%)	Group II n (%)	Group I n (%)	Group II n (%)
0	18(36)	4(8)	6(12)	1(2)
1	18(36)	8(16)	14(28)	1(2)
2	12(24)	9(18)	20(40)	2(4)
3	2(4)	10(20)	8(16)	4(8)
4	0	12(24)	2(4)	13(26)
5	0	5(10)	0	15(30)
6	0	2(4)	0	11(22)
7	0	0	0	2(4)
8	0	0	0	1(2)

When the pain was compared during and after the procedure within the groups, both groups had a p-value <0.05 with significantly less pain three hours after the procedure, which was assessed by the Wilcoxon sign rank test (Table 2).

**Table 2:** Wilcoxon sign rank test showing the mean differences between pain scores during and after the procedure in individual groups * P-value < 0.05 is significant

Wilcoxon sign rank test	Mean rank	Sum of ranks	Z value	P-value
Group I	23.11	716.5	-3.423	0.001*
	16.95	186.5		
Group II	24.21	920	-5.00	0.000*
	11.67	70		

Mann-Whitney U test was done to compare the pain experienced by the subjects between the groups, which proved that during and three hours after the procedure, the group with additional zygomaticotemporal nerve block had significantly lower pain scores (Table 3). 

**Table 3:** Mann-Whitney U test showing mean differences between the pain scores between both the groups in individual timelines * P-value <0.05 is significant

		Mean rank	Sum of ranks	Mann-Whitney U value	P-value
Post OP	Group I	28.72	1436	161	0.000*
	Group II	72.28	3614		
During procedure	Group I	34.1	1705	430	0.023*
	Group II	66.9	3345		

## Discussion

The results of our study prove that zygomaticotemporal nerve block, in addition to supraorbital and supratemporal, has a better effect on controlling the pain experienced by the subjects during platelet-rich plasma mesotherapy in the temporal region. 

The three main layers that make up our skin are the epidermis, dermis (the middle layer), and hypodermis (the layer beneath the skin). The epidermis, the layer of skin that is visible to us, is less than a millimeter thick and has dead cells all over its surface. Dead skin cells continually shed from our skin and are replaced by new epidermal cells that proliferate continuously. Collagen, elastin, and hyaluronic acid-containing connective tissue make up the dermis or middle layer. This region comprises several key skin structures and gives the skin its flexibility. The hair follicle is one such structure. Twenty distinct cell types, each containing one or more hair follicles, make up a follicle. Hair strands are created, develop, and emerge from the skin in hair follicles [16,20-23]. 

The hair shaft is the portion of the hair that is visible. Using a syringe, specific medications and chemicals are injected into the dermis layer during hair mesotherapy. This layer contains the hair follicles; therefore, if we want to deliver the restorative components there effectively, we must send them there first [24]. Mesotherapy, like corticosteroids, is believed to have a wide array of applications, especially in the field of cosmetic dermatology. But the most up-to-date and often used dermatological indications and the medications associated with them are cellulite on the body, lipodissolve, ineffective body contouring skin rejuvenation, pigmentation, lift, shine, male pattern baldness, and telogen effluvium in hair [25-27].

Cellulite, local fat deposits, and face rejuvenation show good outcomes with mesotherapy; telogen effluvium, androgenetic alopecia, stretch marks, and facial pigmentation show moderate results; and body sculpting/contouring and melasma show uncertain results with the same. Topical drugs, such as minoxidil, typically have less of an impact than hair mesotherapy because they don't penetrate the epidermis as well. Several mesotherapy solutions include ingredients that improve blood flow; they include vasodilators, which promote tissue circulation [28,29]. 

Various vitamins, amino acids, and minerals nourish skin cells and follicles in addition to mesotherapy injections. They enhance the strength and condition of the hair by stopping the process of follicle degeneration and regulating sebum secretion. The medication used in mesotherapy regulates sebaceous gland output and amplifies the results of other therapies. In addition to steroid injections, mesotherapy can be used as a supplemental treatment for hair loss and general hair maintenance [30].

In the present study, mesotherapy with platelet-rich plasma (PRP) was used for hair loss. In a similar study, N. Moftah et al. assessed the safety and effectiveness of mesotherapy with a preparation containing dutasteride in treating female pattern hair loss (FPHL) [23]. Group I of this trial received an injection of a preparation containing dutasteride, while Group II received an injection of saline. Patients underwent twelve sessions, and at the eighteenth week, they were assessed by the use of photographs, hair pull tests, patient self-evaluation, and hair diameter measurements. According to the study's findings, mesotherapy using a preparation containing dutasteride was a less intrusive, more successful, and more comfortable treatment option for female pattern hair loss (FPHL) patients, showing superior results over a shorter period of time [23]. 

When treating female pattern hair loss (FPHL), mesotherapy, which consists solely of nutritional supplements, is a more effective, patient-acceptable, and tolerated approach than topical minoxidil [24]. The effectiveness and safety of injecting a 0.05% dutasteride-containing fluid were compared to injecting 0.9% saline using the nappage technique by Marwa et al. At weeks zero, one, two, three, five, seven, and 11, patients received seven injections; at week 12, they underwent evaluation. Compared to the placebo, the dutasteride-containing formulation was noticeably easier to use. Three types of assessment were used to demonstrate this: the difference in hair count, the assessment by independent professionals, and the self-assessment of the patients. The response to mesotherapy was higher the shorter the male pattern hair loss (MPHL) duration. There were very few adverse effects, only minor headaches and pain. Other than these, many studies support mesotherapy for hair loss [23-26]. 

In the present study, patients who received zygomaticotemporal block additionally had significantly less pain during and after three hours of the treatment. The patients who received supraorbital and supratrochlear treatments had comparatively more pain. The reason is that both of these regional blocks don't cover the temporal region. Different sensory nerves correspond to different parts of the scalp. The auriculotemporal branch of the mandibular nerve, the zygomaticotemporal branch of the maxillary nerve, the supraorbital and supratrochlear branches of the ophthalmic nerve, the lesser occipital nerve and the retro auricular nerve from the third (and second) cervical roots, and the greater occipital nerve from the second cervical root are among them [27-30]. 

One of the two branches of the zygomatic nerve branch of the maxillary division of the trigeminal nerve is the zygomaticotemporal nerve. The temporal region and a tiny section of the forehead supply it with sensitive innervation. The nerve is located in the fossa temporalis, lateral to the orbital margin. In order to target the orbital rim, 1 milliliter of 2% lidocaine is often injected in the middle third of the upper side of the malar branch, with the needle advanced at a 45-degree angle. To prevent migration within the orbit, it is best to never reach the bony rim of the orbit. One milliliter is cautiously injected at the end [27,28]. In the present study, the zygomaticotemporal block was used for hair loss. Similarly, this block was used for different treatments such as headaches, migraine, nerve paralysis, and trigeminal neuralgia [30].

Limitations of the study

The comparison of only three injection blocks might limit the assessment of the effectiveness of the zygomaticotemporal block. Further study endeavors employ a larger sample size and compare diverse injection techniques. For mesotherapy, the decision between injection techniques is based on several variables, such as the intensity of the disease, the area of treatment, and the extent of treatment. 

## Conclusions

In summary, the first group experienced substantially less pain throughout the platelet-rich plasma (PRP) therapy and three hours after the operation, according to the results, which showed a significant difference in pain levels between the two groups. The first group's use of the zygomaticotemporal block not only made the treatment more comfortable for the patients but also provided insight into the possible wider uses of this block in other medical procedures. The zygomaticotemporal block has been shown to be useful in lowering pain during PRP treatment for hair loss; hence, it may be investigated further in the treatment of trigeminal neuralgia and headaches.
